# Effect of peer-mother interactive programme on prevention of mother-to-child HIV transmission outcomes among pregnant women on anti-retroviral treatment in routine healthcare in Dar es Salaam, Tanzania

**DOI:** 10.1371/journal.pgph.0000256

**Published:** 2022-03-09

**Authors:** Goodluck Willey Lyatuu, Helga Naburi, Shally Mwashemele, Peter Lyaruu, Roseline Urrio, Brenda Simba, Emmanuel Philipo, Ayoub Kibao, Deborah Kajoka, David Sando, Nicola Orsini, Gunnel Biberfeld, Charles Kilewo, Anna Mia Ekström

**Affiliations:** 1 Management and Development for Health, Dar es Salaam, Tanzania; 2 Department of Global Public Health, Karolinska Institutet, Stockholm, Sweden; 3 Department of Obstetrics and Gynaecology, Muhimbili University of Health and Allied Sciences, Dar es Salaam, Tanzania; 4 Department of Paediatrics and Child Health, Muhimbili University of Health and Allied Sciences, Dar es Salaam, Tanzania; 5 Health Section, United Nations Children’s Fund, Dar es Salaam, Tanzania; 6 Department of Health and Social Welfare, Regional Administrative Secretary, Dar es Salaam, Tanzania; 7 Department of Preventive Services, Tanzania Ministry of Health, Community Development, Gender, Elderly and Children, Dodoma, Tanzania; Babcock University, NIGERIA

## Abstract

Peer support services are increasingly being integrated in programmes for the prevention of mother-to-child HIV transmission (PMTCT). We aimed to evaluate the effect of a peer-mother interactive programme on PMTCT outcomes among pregnant women on anti-retroviral treatment (ART) in routine healthcare in Dar es Salaam, Tanzania. Twenty-three health facilities were cluster-randomized to a peer-mother intervention and 24 to a control arm. We trained 92 ART experienced women with HIV to offer peer education, adherence and psychosocial support to women enrolling in PMTCT care at the intervention facilities. All pregnant women who enrolled in PMTCT care at the 47 facilities from 1^st^ January 2018 to 31^st^ December 2019 were identified and followed up to 31^st^ July 2021. The primary outcome was time to ART attrition (no show >90 days since the scheduled appointment, excluding transfers) and any difference in one-year retention in PMTCT and ART care between intervention and control facilities. Secondary outcomes were maternal viral suppression (<400 viral copies/mL) and mother-to-child HIV transmission (MTCT) by ≥12 months post-partum. Analyses were done using Kaplan Meier and Cox regression (ART retention/attrition), generalized estimating equations (viral suppression) and random effects logistic regression (MTCT); reporting rates, proportions and 95% confidence intervals (CI). There were 1957 women in the peer-mother and 1384 in the control facilities who enrolled in routine PMTCT care during 2018–2019 and were followed for a median [interquartile range (IQR)] of 23 [10, 31] months. Women in both groups had similar median age of 30 [IQR 25, 35] years, but differed slightly with regard to proportions in the third pregnancy trimester (14% versus 19%); advanced HIV (22% versus 27%); and ART naïve (55% versus 47%). Peer-mother facilities had a significantly lower attrition rate per 1000 person months (95%CI) of 14 (13, 16) versus 18 (16, 19) and significantly higher one-year ART retention (95%CI) of 78% (76, 80) versus 74% (71, 76) in un-adjusted analyses, however in adjusted analyses the effect size was not statistically significant [adjusted hazard ratio of attrition (95%CI) = 0.85 (0.67, 1.08)]. Viral suppression (95%CI) was similar in both groups [92% (91, 93) versus 91% (90, 92)], but significantly higher among ART naïve women in peer-mother [91% (89, 92)] versus control [88% (86, 90)] facilities. MTCT (95%CI) was similar in both groups [2.2% (1.4, 3.4) versus 1.5% (0.7, 2.8)]. In conclusion, we learned that integration of peer-mother services in routine PMTCT care improved ART retention among all women and viral suppression among ART naïve women but had no significant influence on MTCT.

## Introduction

Use of lifelong anti-retroviral treatment (ART) for all pregnant and breastfeeding women with HIV regardless of immunologic or clinical disease status, also known as the WHO Option B+ recommendation of 2012 [1], has been a major game changer in the efforts to end the HIV epidemic in children. Use of lifelong ART is known to reduce mother-to-child transmission of HIV (MTCT) to less than 5% in breastfeeding populations and less than 2% in non-breastfeeding populations [1]. Later on, in 2015, the WHO recommended universal ART coverage to all people living with HIV [2], which provided further momentum in efforts to end the AIDS epidemic. Nevertheless in 2020 the estimates of MTCT in the 21 focus countries in Africa with the highest burden of the HIV epidemic, remained unacceptably high at 10.4%, despite 89% ART coverage among pregnant women with HIV [3]. Barriers to successful Prevention of MTCT (PMTCT) in women on lifelong ART include: delayed start of antenatal care (ANC), late HIV diagnosis/enrolment to ART care, incident HIV infections during pregnancy/breastfeeding, sub-optimal ART adherence, attrition from ART care, and low uptake of final infant HIV testing [3]. In particular, challenges of retention and adherence to ART have been among long-standing concerns ever since the roll-out of lifelong ART for all pregnant women with HIV for PMTCT and later all people living with HIV [4–9]. These concerns were driven, in part, by the uncertainty as to whether pregnant women diagnosed with asymptomatic HIV during routine HIV testing as part of ANC, would have the motivation to adhere to lifelong ART and remain in care long-term [6–8]. Retention (here defined as attendance within 90 days of appointment) as low as 76% at 12 months has been reported in a meta-analysis of 22 studies, conducted in the era of lifelong ART for all women in PMTCT care, across 8 African countries involving 60,890 women [4]. This meta-analysis also highlighted low retention two to three years after enrolment in PMTCT care ranging from 41% to 74%, in studies that had two or more years of follow-up, a period that corresponds to the end of PMTCT follow-up [4].

Peer support services have been widely in use to mitigate barriers of uptake, adherence and outcomes in HIV and PMTCT services in low- and middle-income countries [10–12]. The motivation for peer support services has been partly driven by challenges of human resource shortages in understaffed overburdened health care systems, and also the demand for counselling, adherence and psychosocial support among women receiving PMTCT care [10, 13–17]. Although there is rich qualitative literature on the engagement of peer-mothers in PMTCT care [14, 18–20], the literature remains scarce on quantitative evaluation of the effect of peer-mothers on PMTCT outcomes, specifically ART adherence and retention, viral suppression and MTCT, in the era of lifelong ART for PMTCT [10–12, 21]. Furthermore, few available studies indicate mixed results with some highlighting modest benefit and others reporting non-statistically significant effect of peer-mother engagement on PMTCT outcomes [22–24]. Our study, therefore, aimed to evaluate the effect of a peer-mother interactive programme, involving innovative peer support services, to address challenges of retention and adherence to ART and MTCT among women on lifelong ART for PMTCT in routine healthcare settings.

## Methods

### Ethics statement

This study was approved by the ethical review boards of Muhimbili University of Health and Allied Sciences, ref. 2017-06-28/AEC/Vol.XII/83, and the Tanzania National Institute for Medical Research, ref. NIMR/HQ/R.8a/Vol.IX/2594. Informed consent was waived on the basis that the study used anonymized data from electronic routine healthcare records.

### Study design and setting

**Fig 1:**
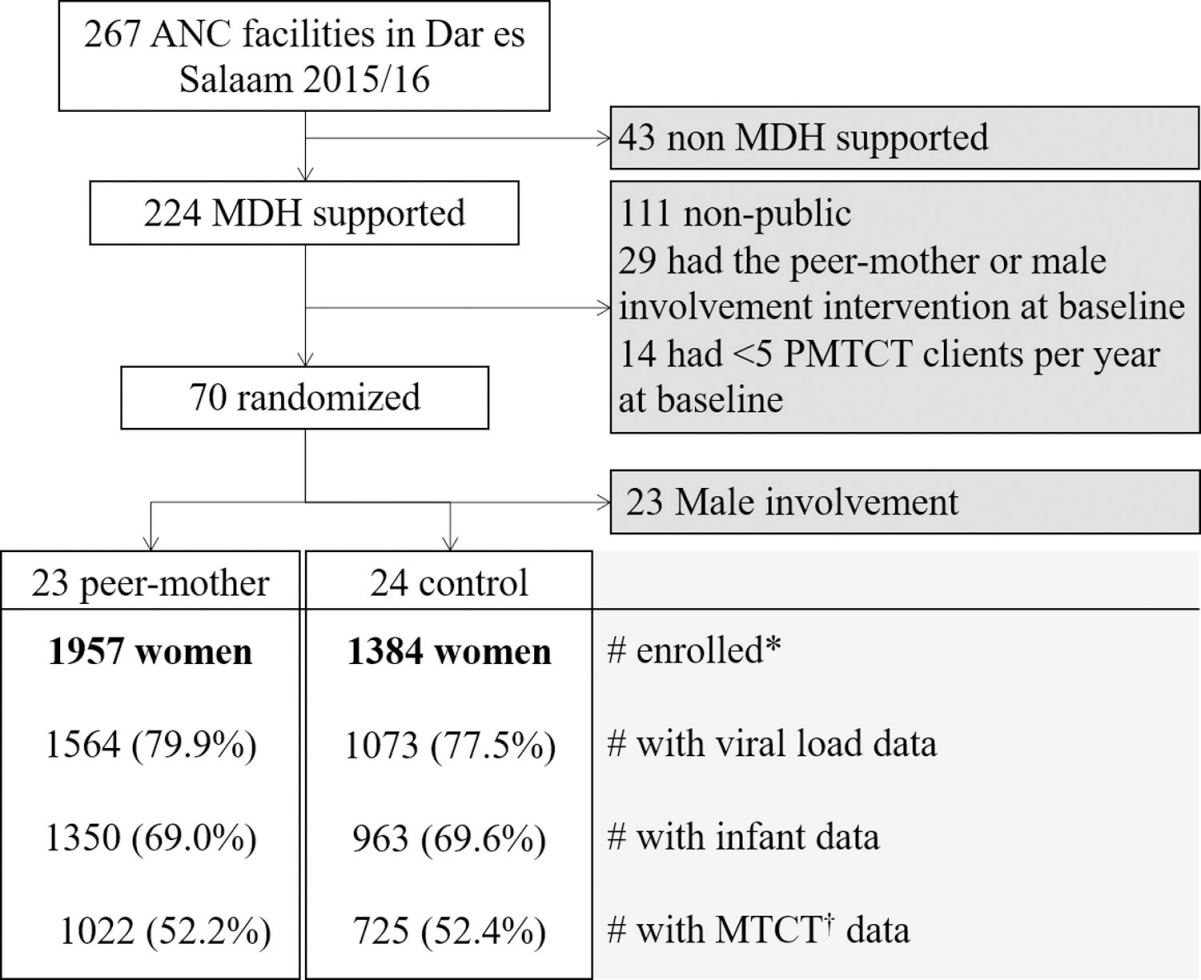
Study flow diagram. *Enrolment period = 1^st^ January 2018 to 31^st^ December 2019; Follow-up to 31^st^ July 2021. ^†^MTCT status by 12 or more months post-partum. Abbreviations: PMTCT = prevention of mother-to-child transmission of HIV, MTCT = mother-to-child transmission of HIV.

We conducted a cluster randomized implementation study in routine healthcare settings (providing day-to-day clinical PMTCT care) of Dar es Salaam, Tanzania. Dar es Salaam is the largest and main commercial city of Tanzania with a population of over five million people and an adult HIV prevalence of 4.3% per the 2016/17 Tanzania HIV impact survey [25, 26]. During the study period, the city was providing ART care to about 180,000 people living with HIV and registered about 170,000 new pregnant women in ANC services annually with an HIV positivity among ANC attendees of 5.0% in 2018/19 [27]. Our study capitalized on and was embedded in the planned scale-up of two interventions–peer-mother adherence support and male involvement in ANC/PMTCT services–that had been implemented as part of efforts to improve PMTCT outcomes in routine care across 29 public health facilities in Dar es Salaam but their impact had not been adequately assessed. This scale-up was led by the Management and Development for Health (MDH), a non-governmental organization supporting HIV prevention care and treatment services in 224 health facilities in Dar es Salaam at that time, and across Tanzania. A sub-set of 70 public health facilities with at least 5 clients in PMTCT care between 1^st^ October 2015 and 30^th^ September 2016 was identified (Fig 1) for the scale-up and this evaluation study. A computer-generated random sequence in Stata was used to block-randomize these 70 facilities to 23 peer-mother, 23 male involvement (not part of this paper) and 24 control facilities (Fig 1). Randomization blocks used were: administrative districts (Ilala, Kinondoni and Temeke) and PMTCT client volume (less than 20 versus 20 or more women) at that time.

In Tanzania, PMTCT is integrated with ANC and provided under one roof as standard of care. All pregnant women found to be HIV positive at ANC, are enrolled in PMTCT care and started on lifelong ART on the day of HIV diagnosis, or, those with known HIV are encouraged to continue taking their ART. Since 2019, the default adult (including pregnant women) first-line ART regimen backbone in Tanzania changed to an integrase inhibitor-based regimen containing dolutegravir (DTG). Patients who were on the first-line ART containing non-nucleoside reverse transcriptase inhibitor (NNRTI) were gradually transitioned to DTG regimens from 2019 and onwards. Protease inhibitors (PIs) are backbone regimens for second-line ART. All PMTCT clients are followed up through monthly ANC-PMTCT visits for: clinical and laboratory monitoring; ART refills; counselling on adherence, infant feeding and Early Infant Diagnosis of HIV (EID); and other antenatal/ postnatal care services. As part of routine care, women who miss their scheduled PMTCT follow-up visit by three or more days are traced via phone call by a health care provider (HCP) and, when unsuccessful, a home visit by a community health worker (CHW). These women are declared as lost to follow-up after at least three unsuccessful tracking efforts over 90 days. Viral load monitoring is done at first ANC visit for women already on ART, and three months after starting ART for women new on ART, then six-monthly until cessation of breastfeeding, and thereafter once annually. Women with high viral loads (1000 or more copies per mL) are offered enhanced adherence counselling, repeat viral load testing after three months and are switched to second line ART upon persistent high viral load. At birth, infants born to women with HIV, i.e. HIV Exposed Infants (HEIs) are given daily nevirapine prophylaxis for six weeks, with addition of zidovudine for high risk HEI (born to mothers on ART for less than four weeks or with high viral load). At six weeks, HEI receive their first HIV test for EID using DNA-polymerase chain reaction (PCR), started on daily co-trimoxazole prophylaxis and enrolled on monthly mother-infant follow-up. Follow-up for the mother and infant in PMTCT care goes on for up to two years post-partum unless the infant is diagnosed with HIV infection, at which point the infant and mother are transitioned to routine ART care. Infants who are not HIV infected are discharged after a negative HIV antibody test by 18 months of age. All HIV patients in Tanzania, are issued national HIV care and treatment clinic (CTC) unique identification (ID) numbers, known as CTC-ID. The CTC-ID is used to register, record and manage ART/ PMTCT care of all HIV patients using health facility charts (CTC2 cards) and the national electronic CTC2 database, as well as identify/link patients across facilities upon transfer of care. The HEI are also issued unique IDs and registered in HEI cards, linked to their mother’s CTC-ID, which is used to record and manage their care including EID tests.

**The study population** included all pregnant women with HIV (new, previously diagnosed, and, transfers), and later their infants, enrolling in routine PMTCT care at peer-mother and control facilities between 1^st^ January 2018 to 31^st^ December 2019, in total 1957 women in peer-mother facilities and 1384 women in control facilities (Fig 1). Study participants were followed up until 31^st^ July 2021 with a median [interquartile range (IQR)] follow up of 23 [10, 31] months in both groups.

### Description of the intervention

The peer-mother interactive programme was built upon the foundations of the NAMWEZA approach, pioneered by researchers in Uganda and Tanzania [28]. NAMWEZA (a term derived from Swahili words meaning “Yes, together we can”) is a peer-based participatory approach built on the framework and principles of appreciative inquiry, which entails the use of appreciative conversations and relationships to influence attitudes, behaviours and practices [28, 29]. The NAMWEZA approach employs appreciative conversations, clarification of values, strengthening assertiveness, reflection and envisioning of the future to: stimulate dialogue; impart knowledge; restore self-esteem and self-efficacy; reinforce a positive attitude towards health and life and empower persons with HIV to become positive change agents to their peers and in the community [28]. The approach also draws upon Albert Bandura’s social cognitive theory, which describes the dynamic interaction between an individual, her/his environment and behaviour, and how self-efficacy and social structural support influences sustainable behaviour change in this context [30]. Our study hypothesized that the NAMWEZA-trained peer-mothers could be employed to mitigate barriers that hinder optimal ART adherence, retention and PMTCT among pregnant women with HIV enrolling in PMTCT care, such as HIV stigma, inadequate motivation to adhere to lifelong ART and inadequate psychosocial support.

At baseline, in July 2017, inception meetings were conducted with local government health management teams and HCPs from the 23 peer-mother facilities to sensitize and orient key stakeholders on the intervention. These HCPs were thereafter asked to recruit two to ten potential peer-mother candidates per intervention facility from among experienced PMTCT clients who had been on ART for at least six months and were willing and able to share their experience with others. A total of 180 women were recruited and trained (in groups of 15 to 40 women) on the basics of HIV, ART, PMTCT and peer-mother support services using the NAMWEZA approach and trainers. The trainings took place from August 2017 to February 2018 and comprised of ten sessions spaced out once weekly for ten weeks to allow time for reflection after each session. After training, 92 peer-mothers (one to eight per facility) who demonstrated good comprehension and competency on the peer-mother services during the training, were deployed back to their facilities to implement the peer-mother initiative (as each respective group completed the 10 weeks of training) starting from January 2018. Deployed peer-mothers were given a monthly stipend for transport and communication of about USD 65, equivalent to the average stipend rate for lay cadres at MDH and across Tanzania, to support them to attend their PMTCT clinics at least two to three days per week for peer-mother services. Peer-mothers were attached to a focal PMTCT HCP at her clinic for onsite mentorship and support, and were visited at least once monthly for continued mentorship, supervision and support at least in the first year of the peer-mother intervention. At the PMTCT clinics, peer-mothers offered health education on ART/PMTCT care and reached out to new PMTCT clients for individualized dialogue, health education, adherence support and follow-up. The dialogues focused on assisting new PMTCT clients to understand and accept their HIV status, identify and address stigma, restore self-esteem and self-value, prepare to safely disclose HIV status to their partners, and provide adherence support as needed. The peer-mothers also facilitated creation of seven peer support groups for women living with HIV that met once to three times quarterly to share experience and educate each other on PMTCT and peer support services. At these peer support groups peer-mothers also collaborated with each other in small group savings and lending services, commonly known as village community bank (VICOBA) to support each other on socio-economic needs/activities. During the course of the study, 10% to 20% of peer-mothers dropped-out per year, mainly due to transferring care or opting out of peer-mother services, hence replacements and optimization was done with a focus towards having a smaller skilled and efficient team of peer-mothers. The optimization was done by strengthening follow-up, mentorship and monitoring of the peer-mother’s work and deliverables. Furthermore, during the period of April to October 2020, in response to the first wave of the COVID19 pandemic, peer-mother services were temporarily suspended and resumed in November 2020. Therefore, at study endpoint in July 2021 a total of 35 peer-mothers remained active within the peer-mother intervention across the 23 study facilities.

**At control facilities** standard of care was provided which entailed routine PMTCT care described above, with adherence support offered by HCPs and tracing of clients who miss appointment by HCPs and CHWs.

**The primary study outcome** was time to ART attrition from the day of enrolment in PMTCT and ART care in index pregnancy, over the entire three and a half years of follow-up, and the corresponding percent one-year retention in PMTCT and ART care. We defined ART attrition (event of interest) as women who discontinued ART for any reasons including death, stopping ART for more than 90 consecutive days, or loss to follow-up (no show for more than 90 consecutive days since scheduled appointment among non-transfers) at any point during follow-up. One-year retention in PMTCT and ART care was defined as being alive and picking ART within 90 days of scheduled appointment in the first twelve months of PMTCT care, excluding documented transfers. For women who did not have a documented next appointment date, we adjusted our loss to follow-up definition to “no show for more than 180 consecutive days since last clinical/ART refill visit”, based on maximum allowable appointment spacing per national ART guidelines in Tanzania. As secondary outcomes we analysed viral suppression (<400 viral copies per mL) from among women with viral load data and MTCT by 12 months, i.e. infants with a positive HIV test from among mother-infant pairs with an MTCT status by 12 or more months post-partum (i.e. excluding HIV negative infant tests before 12 months of age). All study participants were included in the primary outcome (attrition) analysis whereas the secondary outcome analyses included only women with documented outcomes of interest. Therefore, 1564 (79.9%) women in the peer-mother and 1073 (77.5%) in the control facilities with viral load data were included in the viral suppression analysis and 1022 (52.2%) women in the peer-mother and 725 (52.4%) in the control facilities with data on infant HIV status by 12 months or more, were included in the MTCT outcome analysis (Fig 1).

### Data collection

Study data was extracted from the national electronic CTC2 database. Extracted data included: the woman’s: date of birth; dates of HIV diagnosis, PMTCT enrolment and ART initiation; gestational age, clinical HIV disease stage and CD4 count and ART regimen at PMTCT enrolment; as well as viral load results. Infant data included infant date of birth, follow-up status and EID test result. We also collected data on facility level attributes including annual volume of PMTCT clients, and coverage of couple ANC attendance and HIV testing at ANC.

**Data analysis** followed intention to treat protocol. We used Stata Software version 15 for all our statistical analyses. Continuous variables were summarized by medians and IQR, then categorized based on clinical relevance, and categorical variables were summarized by proportion. We assessed the balance of randomization between intervention and control facilities using the Chi-square test. Time to ART attrition based on a single failure per subject (the primary outcome) and the corresponding proportion of women retained in the first year of PMTCT care were analysed using the Kaplan Meier estimator in unadjusted analysis [31]. Women with documented transfer outside of the study facilities were right censored on the date of their last visit as non-attrition. Women who did not have a follow-up period, i.e. made only one visit or experienced a failure event on their first visit, were assigned a 0.1-day of follow-up so as to retain them in the survival analyses. All participants were censored at the end of follow-up on 31^st^ July 2021.We plotted Kaplan Meier survival (ART retention) curves across months since enrolment in PMTCT care and used the log rank test to make crude comparisons of attrition rates and the proportion of women retained in peer-mother versus control facilities. We then performed un-adjusted sub-group analyses of one-year ART retention by women’s baseline characteristics, using the Kaplan Meier estimator, comparing peer-mother versus control facilities using the log rank test (Table 2). Thereafter, we performed a multivariable Cox proportional hazard regression with shared frailties [31], adjusting for clustering at health facilities and the baseline imbalances after randomization (using all maternal variables in Table 1), to compare adjusted hazards of attrition among women in the intervention versus control facilities (S1 Table). For virological suppression (a secondary outcome) we used binary Poisson generalized estimating equations (GEE) with robust error variance, to compare the proportion of women who were virally suppressed (less than 400 viral copies/mL) on repeated measures of viral load tests over time, in peer-mother versus control facilities. We also performed sub-group analyses of viral suppression by baseline characteristics comparing peer-mother and control facilities using predicted probabilities of viral suppression from bivariable Poisson GEE regression models. Thereafter we performed a multivariable GEE regression of the risk of virologic failure in peer-mother versus control facilities adjusting for baseline imbalances after randomization. For MTCT risk (secondary outcome), we used random effects multivariable logistic regression, adjusting for clustering at health facilities and baseline imbalances after randomization, to compare MTCT risks by 12 months in peer-mother versus control facilities. Across these analyses we report: attrition rate, hazard ratio (HR) of attrition and proportion of women retained; proportion of women virally suppressed; proportion of mother-infant pairs with MTCT and odds ratio (OR) of MTCT; as well as their corresponding 95% confidence intervals (CI).

**Table 1:** Baseline characteristics of pregnant women who enrolled in PMTCT care peer-mother (N = 1957) versus control (N = 1384) health facilities.

Characteristic	Health facility randomization status		P value
	Peer-mother: n (%)	Control: n (%)	
**Patients characteristics**			
Maternal age, years			0·31
<20	76 (3·9)	67 (4·8)	
20–29	868 (44·4)	592 (42·8)	
30–39	904 (46·2)	634 (45·8)	
≥40	109 (5·6)	91 (6·6)	
Gestational age, weeks			0·0055
<13 (first trimester)	365/ 1709 (21·4)	231/ 1172 (19·7)	
13–27 (second trimester)	1097/ 1709 (64·2)	719/ 1172 (61·4)	
≥28 (third trimester)	247/ 1709 (14·5)	222/ 1172 (18·9)	
Advanced HIV disease*			0·0011
no	1520 (77·7)	1007 (72·8)	
yes	437 (22·3)	377 (27·2)	
ART status			<0·0001
On ART before PMTCT care	882 (45·1)	728 (52·6)	
Starting ART at/ after PMTCT care	1075 (54·9)	655 (47·3)	
Did not start ART		1 (0·1)	
ART regimen backbone			0·0033
NNRTI	1770 (90·4)	1296 (93·6)	
PI	65 (3·3)	25 (1·8)	
DTG	122 (6·2)	62 (4·5)	
Did not start ART		1 (0·1)	
Infant sex			0·62
male	704/ 1350 (52·2)	492/ 963 (51·1)	
female	646/ 1350 (47·9)	471/ 963 (48·9)	
**Health facility attributes**			
PMTCT client Volume, women/year			<0·0001
1–12	105 (5·4)	101 (7·3)	
13–60	797 (40·7)	770 (55·6)	
61–363	1055 (53·9)	513 (37·1)	
Percent couple HIV testing at ANC			0·26
<50	1180 (60·3)	861 (62·2)	
≥50	777 (39·7)	523 (37·8)	

*WHO stage III/ IV or CD4 count <200cells/μL; p values were calculated using the χ2 test.

**Abbreviations:** PMTCT = prevention of mother-to-child transmission of HIV; ART = anti-retroviral treatment; NNRTI = non-nucleoside reverse transcriptase inhibitors; PI = protease inhibitors, DTG = dolutegravir. HIV = human immune deficiency virus; WHO = World Health Organization; CD = cluster of differentiation; ANC = antenatal care.

## Results

### Baseline characteristics

Table 1 compares baseline characteristics of the 1957 women in peer-mother versus the 1384 women in control facilities who started PMTCT care between 1^st^ January 2018 to 31^st^ December 2019. Both the women’s age and the proportions of female infants was similar in the peer-mother and control facilities (Table 1). However, among women with data, slightly fewer (14.5%; 247 out of 1172) in peer-mother facilities compared to 18·9% (222 out of 1172) in control facilities were in third trimester of pregnancy. Also, peer-mother facilities had slightly fewer women with advanced HIV i.e. 22·3% versus 27·2% in control facilities, and fewer women who started ART before pregnancy i.e. 45·1% versus 52·6% in control facilities.

### Retention in PMTCT care

By the end of the study follow-up period, 305 (15.6%) women in peer-mother versus 218 women (15.8%) in control facilities had transferred out to other facilities, and ART attrition occurred in 543 (27.7%) women in the peer-mother versus 453 (32.7%) in the control facilities. A major driver of ART attrition was loss to follow-up, occurring in 515 (26.3%) women in peer-mother and 437 (31.2%) women in control facilities. The unadjusted attrition rate (95%CI) per 1000 person months over the entire 3.5 years of follow-up was lower in peer-mother facilities [14.3 (13.1, 15.5)] compared to control facilities [17.6 (16.0, 19.3)], unadjusted HR (95%CI) = 0.82 (0.67, 0.94). The attrition rate per 1000 person months in peer-mother versus control facilities had a more pronounced difference among ART naïve women [22.4 versus 32.0, p = 0.0005] than ART experienced women [7.5 versus 9.9, p = 0.011].

**Fig 2:**
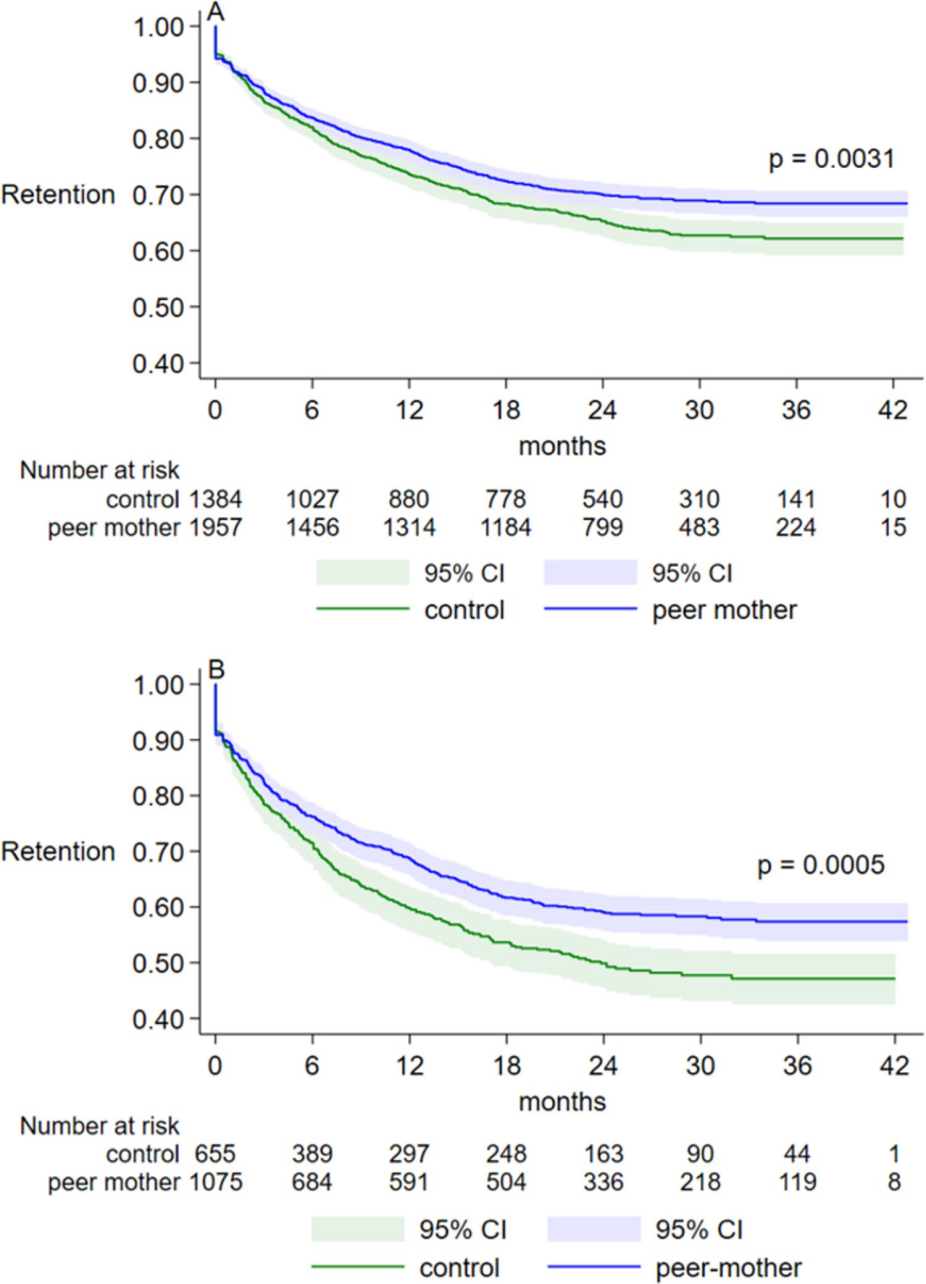
Retention on ART across months since PMTCT enrolment. Kaplan Meier survival analysis with failure (ART attrition) defined as death, opting out or loss to follow-up (no show >90 days since previous scheduled appointment) for (A) all women and (B) ART naïve women at baseline, p-value computed using log rank test. Abbreviations: ART = anti-retroviral treatment; PMTCT = prevention of mother-to-child transmission of HIV; CI = confidence intervals.

In the Kaplan Meier survival analysis, retention on ART in the first year of PMTCT care was significantly higher among women in peer-mother (78.0%) than control (73.6%) facilities, (Fig 2A and Table 2). The effect of the peer-mother intervention on ART retention was more pronounced among women who were ART naïve at baseline (Fig 2B), resulting in 9.1 percentage points higher retention in the first year of PMTCT care in peer-mother versus control facilities whereas among ART experienced women the effect size was 3.3 percentage points (Table 2). Significantly higher one-year retention in peer-mother compared to control facilities was also observed in sub-groups of women aged 30–39 years old, women with early stage HIV disease and women on NNRTI regimen (Table 2). In the adjusted multivariable Cox regression (S1 Table), the hazard of ART attrition was lower but non-statistically significant in peer-mother compared to control facilities, adjusted HR (95%CI) = = 0.85 (0.67, 1.08).

**Table 2:** Retention on ART in the first year of PMTCT care among women in peer-mother (N = 1957) versus control (N = 1384) facilities.

Baseline characteristic	Health facility randomization status		
	Peer-mother: % (95%CI)	Control: % (95%CI)	P value
**Patients characteristics**			
Maternal age, years			
<20	59.7 (46.5, 70.7)	59.1 (45.2, 70.6)	0.86
20–29	71.9 (68.6, 74.9)	67.8 (63.6, 71.6)	0.16
30–39	84.1 (81.5, 86.4)	78.7 (75.3, 81.8)	0.014
40+	84.5 (75.9, 90.2)	84.0 (74.5, 90.2)	0.78
Gestational age, weeks			
<13 (first trimester)	83.1 (78.7, 86.7)	80.7 (74.7, 85.4)	0.65
13–27 (second trimester)	78.1 (75.5, 80.5)	74.0 (70.5, 77.1)	0.058
≥28 (third trimester)	74.5 (68.3, 79.7)	74.2 (67.7, 79.5)	0.98
Advanced HIV disease*			
no	75.5 (73.2, 77.7)	68.7 (65.6, 71.6)	0.0009
yes	86.1 (82.4, 89.1)	85.7 (81.7, 88.9)	0.80
ART status			
On ART before PMTCT care	88.4 (86.1, 90.4)	85.1 (82.3, 87.5)	0.050
Starting ART at/ after PMTCT care	68.8 (65.7, 71.6)	59.7 (55.5, 63.6)	0.0010
ART regimen backbone			
NNRTI	77.5 (75.4, 79.4)	72.9 (70.3, 75.3)	0.011
PI	93.5 (83.7, 97.5)	87.0 (64.8, 95.6)	0.30
DTG	76.8 (68.1, 83.5)	83.9 (72.1, 91.0)	0.30
**Health facility attributes**			
PMTCT client volume, women/ year			
1–12	68.1 (57.8, 76.4)	59.6 (48.9, 68.8)	0.24
13–60	75.4 (72.1, 78.4)	70.4 (66.9, 73.7)	0.052
61–363	80.8 (78.2, 83.1)	80.7 (76.9, 83.9)	0.99
Percent couple HIV testing at ANC			
<50	78.1 (75.5, 80.4)	73.9 (70.7, 76.8)	0.054
≥50	77.7 (74.5, 80.6)	73.3 (69.1, 77.0)	0.079

*WHO stage III/ IV or CD4 count <200cells/μL; p values derived from the log rank test.

**Abbreviations:** PMTCT = prevention of mother-to-child transmission of HIV; ART = anti-retroviral treatment; NNRTI = non-nucleoside reverse transcriptase inhibitors; PI = protease inhibitors, DTG = dolutegravir. HIV = human immune deficiency virus; WHO = World Health Organization; CD = cluster of differentiation; ANC = antenatal care.

### Viral suppression

**Fig 3:**
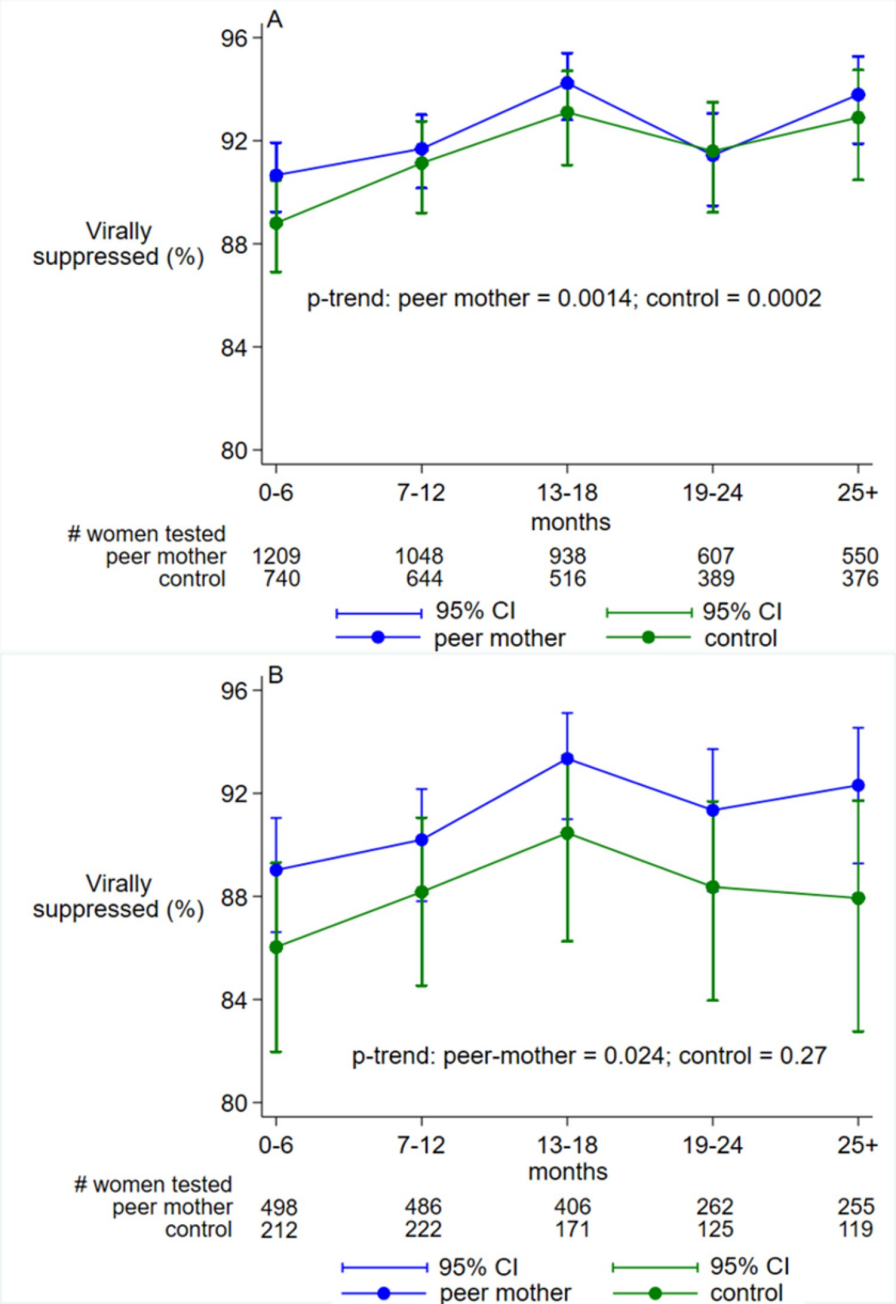
Viral suppression on repeated measures of viral load tests across months since PMTCT enrolment. Binary Poisson generalized estimating equations with robust error variance, with viral suppression defined as <400 viral copies per mL, for (A) all women [N = 1564 in peer-mother and 1073 in control facilities] and (B) ART naïve women at baseline [N = 740 in peer-mother and 393 in control facilities]. Abbreviations: PMTCT = Prevention of mother-to-child transmission of HIV; CI = Confidence Intervals.

A total of 1564 (79.9%) women in the peer-mother facilities and 1073 (77.5%) women in the control facilities had at least one viral load test done during the study follow-up period. Overall, viral suppression among all women with data was high but similar in both peer-mother and control facilities [92.0% versus 91.1%, p = 0.18). Viral suppression increased significantly across months from PMTCT enrolment in both groups ranging from 90.7% at 0–6 months to 93.8% at ≥25 months in peer-mother facilities and 88.8% at 0–6 months to 92.9% at ≥25 months in control facilities (Fig 3A). However, among pregnant women with a newly diagnosed HIV infection who were ART naïve pregnant women, viral suppression (95%CI) was significantly higher in the peer-mother facilities [90.8% (89.3, 92.1)] than in the control facilities [88.1% (85.7, 90.1)]. In this sub-group, only women in peer-mother facilities exhibited a significant increase in viral suppression over time, but not in control facilities (Fig 3B).

Of note, from the 13–18 months timepoint to 19–24 months post PMTCT enrolment viral suppression dropped in both peer-mother facilities (from 94.2% to 91.4%) and control facilities (from 93.1% to 91.6%), Fig 3A. Similarly, among ART naïve women, viral suppression also dropped from 93.3% to 91.3% in peer-mother facilities and from 90.4% to 88.4% in control facilities (Fig 3B). This timepoint corresponded to the period during and after the six months temporary suspension of peer-mother services in response to the first wave of COVID19 pandemic. On sub-group analysis by baseline characteristics, other than the sub-group of ART naïve women, none of the other sub-groups had significant differences in proportion of women virally suppressed in peer-mother versus control facilities (Table 3). In the adjusted multivariable Poisson GEE regression, the adjusted risk ratio of virologic failure in the peer-mother facilities versus control facilities was 0.96 (95%CI: 0.78, 1.19).

**Table 3:** Viral suppression on repeated measures of viral load tests across months since PMTCT enrolment in peer-mother (N = 1957) versus control (N = 1384) facilities.

Baseline characteristic	Health facility randomization status		
	Peer-mother: % (95%CI)	Control: % (95%CI)	P value
**Patients characteristics**			
Maternal age, years			
<20	88.5 (80.6, 93.4)	91.8 (84.5, 95.8)	0.43
20–29	90.0 (88.4, 91.4)	88.2 (85.9, 90.2)	0.17
30–39	93.8 (92.6, 94.8)	92.7 (91.1, 94.0)	0.22
40+	92.8 (89.1, 95.3)	95.5 (91.7, 97.6)	0.21
Gestational age, weeks			
<13 (first trimester)	92.2 (89.9, 94.0)	92.4 (89.6, 94.5)	0.89
13–27 (second trimester)	92.0 (90.7, 93.1)	91.0 (89.2, 92.5)	0.32
≥28 (third trimester)	92.0 (89.3, 94.1)	92.1 (89.0, 94.4)	0.95
Advanced HIV disease*			
no	91.9 (90.8, 92.9)	90.5 (89.0, 91.9)	0.12
yes	92.3 (90.5, 93.8)	92.1 (89.9, 93.8)	0.85
ART status			
On ART before PMTCT care	93.1 (91.9, 94.2)	92.7 (91.2, 93.9)	0.64
Starting ART at/ after PMTCT care	90.8 (89.3, 92.1)	88.1 (85.7, 90.1)	0.032
ART regimen backbone			
NNRTI	92.0 (91.0, 92.9)	91.1 (89.8, 92.2)	0.24
PI	87.2 (81.9, 91.0)	82.1 (71.5, 89.4)	0.29
DTG	96.7 (93.1, 98.4)	96.6 (88.8, 99.0)	0.97
**Health facility attributes**			
PMTCT client Volume, women/year			
1–12	83.5 (78.0, 87.8)	84.8 (78.3, 89.7)	0.72
13–60	90.2 (88.6, 91.7)	90.1 (88.2, 91.6)	0.87
61–363	94.0 (92.9, 95.0)	93.2 (91.4, 94.6)	0.37
Percent couple HIV testing at ANC			
<50	92.8 (91.7, 93.9)	91.1 (89.5, 92.5)	0.064
≥50	90.8 (89.2, 92.2)	90.9 (88.8, 92.2)	0.92

*WHO Stage III/ IV or CD4 count <200cells/μL; p values derived from binary Poisson generalized estimating equations regression.

**Abbreviations:** PMTCT = prevention of mother-to-child transmission of HIV; ART = anti-retroviral treatment; NNRTI = non-nucleoside reverse transcriptase inhibitors; PI = protease inhibitors, DTG = dolutegravir. HIV = human immune deficiency virus; WHO = World Health Organization; CD = cluster of differentiation; ANC = antenatal care.

### MTCT by 12 or more months postpartum

A total of 20 out of 1022 infants in the peer-mother facilities and 9 out of 725 infants in the control facilities who had a confirmed HIV status by at least 12 months of age were diagnosed with HIV infection. This yielded low and similar un-adjusted MTCT risks in both groups: 2.2% (95%CI: 1.4 to 3.4) in the peer-mother and 1.5% (95%CI: 0.7 to 2.8) in the control facilities; p = 0.31. A majority of HIV positive infants in both the peer-mother (16 out of 20) and control facilities (6 out of 9) were diagnosed already by 2 months of age. In the adjusted multivariable random effects logistic regression, the risk of MTCT in the peer mother facilities compared to control facilities remained similar, adjusted OR (95%CI) = 1.87 (0.69, 5.08).

## Discussion

This cluster-randomized study examined the effect of integrating peer adherence and psychosocial support services to improve PMTCT outcomes in pregnant women on lifelong ART care in routine healthcare settings in Tanzania. We found that integration of peer-mother support services resulted in significantly lower ART attrition rate (14 versus 18 per 1000 person months) over three and a half years of follow-up and a modest but significantly higher one-year retention in PMTCT and ART care (78% versus 74%) in unadjusted analyses. Although the retention in PMTCT and ART care was still far from the ideal ≥ 90% even in the intervention facilities and not significant in adjusted analysis, this study provides useful insights regarding the potential of intensifying the role of peer-mothers in mitigating attrition from lifelong ART among women living with HIV during and after PMTCT care. Peer-mothers can be particularly complementary in overburdened, understaffed and resource constrained care settings that are struggling to retain women on ART care, especially post-partum, a common challenge as we move toward universal lifelong ART for all people living with HIV. Of note, the tendency towards higher benefits of the peer-mother intervention among ART naïve women and among those in early stages of their HIV disease, indicates that peer-mother support may be particularly important to mitigate retention barriers among newly diagnosed asymptomatic women who have a higher risk of poor retention [4].

Our findings add to a growing body of evidence that reaffirms the contribution of peer-mother services in improving PMTCT outcomes. Only three previous publications (one cluster randomized and two cohort studies) were identified that quantitatively evaluated the effect of peer-mother services on ART retention, viral suppression and MTCT in the era of lifelong ART initiation during PMTCT [22, 23, 32]. A Malawian 3-arm cluster randomized study found higher two-year ART retention among women who received facility-based (80%) or community-based (83%) peer support services compared to that in standard-of-care control facilities (66%) [23]. Similarly, a Nigerian cohort study, found higher retention by six months post-partum (62%) among women who received care in health facilities with structured peer-mother support services compared to those in standard-of-care (25%) [32]. Conversely, a Malawian study comparing three interventions in routine healthcare settings, observed higher PMTCT attrition following a facility-based mentor-mother (another term for peer-mothers) intervention compared to support from community-based expert clients and CHW interventions [22]. However, in that study researchers compared three fairly similar interventions that engaged lay cadres to support follow-up and retention in PMTCT care, of which the mentor-mothers had multiple other roles unlike the community-based expert clients and CHWs [22]. The growing evidence in support of peer-mother interventions, highlights an opportunity for targeted optimization of this intervention to specifically address barriers and optimize ART retention during and after PMTCT. This includes increasing the frequency, intensity and quality of peer-mother interactions with PMTCT clients who are newly HIV-diagnosed, early in their HIV disease and adolescents, sub-groups found to have the highest risk of poor retention in our data, but also those who seem to benefit the most from this intervention.

Our study found significantly higher viral suppression among the newly HIV-diagnosed ART naïve women in peer-mother (91%) versus control (88%) facilities. This implies that peer-mothers can support known challenges of newly HIV-diagnosed women in PMTCT care such as adjusting to one’s new HIV status, HIV anxiety and stigma, disclosure of HIV status, obtaining social support as well as adapting to consistent use of lifelong medication [14, 33, 34].

Overall, among all women studied, we observed high and similar proportions of viral suppression (< 400 viral copies/mL) and significant positive trends of improved viral suppression over time in care in both arms. This may be attributed to successful roll-out of lifelong ART for all women in PMTCT care, transition to newer integrase inhibitor-based ART regimens from 2019, improved overall quality of care and ART adherence among women who remain in care, as reported earlier from a similar setting [35].

Our study was directly affected by COVID19 that caused a temporary suspension of peer-mother services for at least six months due to safety concerns in response the first wave of the pandemic in Tanzania from March 2020. The COVID19 pandemic, and countries’ response to it, has had multiple health and social impacts including loss of lives, disruption of livelihoods and deterred access and/or uptake of health services in Tanzania and the world at large [36, 37]. Of note, the observed drop in the proportion of women virally suppressed that followed the suspension of peer-mother services around months 19–24 of follow-up (Fig 3A and 3B) recovered after resumption of these services, which further reaffirms the benefit of peer-mother services.

The three previous studies of peer-mother interventions in Africa mentioned above, reported mixed findings on virologic outcomes in PMTCT care. In the Malawian cohort study, the mentor mother intervention was associated with a higher (90%) viral suppression (<1000 viral copies/mL) than the expert clients (78%) intervention [22], indicating, although not clearly explained, that adherence support by women with similar prior PMTCT experiences may be more effective than that from expert clients. The other Malawian cluster randomized study which enrolled only ART naïve women, showed similar high proportions of women virally suppressed (<1000 viral copies/mL) at 2-years ranging from 94% to 96% across all three study arms [23], but lower viral load testing uptake in control (81%) compared to the intervention (91–94%) facilities, implying a possible selection bias. An even more pronounced difference, and potential bias in the uptake of viral load testing (79% in intervention versus 21% in control facilities) was observed in the Nigerian study, rendering the comparison in viral suppression between study arms and between studies difficult [32].

On the MTCT outcome, the low and similar proportions of MTCT by 12 months among women in both peer-mother (2.2%) and control (1.5%) facilities observed in our study as well as in Malawi [23], is gratifying and consistent with high viral suppression across both groups, signifying the success of introducing life-long ART for all in PMTCT care in Africa.

Our reliance on data from routine healthcare settings has several implications. In the absence of a robust evaluation of how peer-mothers adhered to the key components of the intervention, suboptimal implementation in some facilities is possible and would, if being the case, underestimate the true impact of the intervention. Furthermore, the attrition of peer-mothers over time as well as the suspension of their services due to COVID19, could also contribute to blunt the effect size of the intervention. Other concurrent routine care efforts to strengthen PMTCT and ART services—including transition to DTG regimens, improved couple HIV testing at ANC, and improved follow-up and tracking of ART patients—may have improved PMTCT outcomes overall, further diminishing the difference between control and peer-mother facilities. Overall, we believe that the routine care setting is a major strength of our study representing realities, easily transferable to other real-life PMTCT settings in sub-Saharan Africa. The fact that we relied entirely on routine care data for all our analyses minimises the risk of bias due to study procedures. Furthermore, missing data and incomplete follow-up, which inherently is a problem in real-life routine study designs, was non-differential across intervention and control facilities in our study (Fig 1) and therefore less likely to influence interpretation of our findings.

## Conclusion

Our study contributes to a growing body of evidence on the effectiveness of peer-mother support services in improving ART retention among women in PMTCT care, as well as viral suppression among ART naïve women. Peer-mothers could play an important role to further optimize ART for life among women who are newly diagnosed with HIV, in early stages of HIV and among adolescents. These findings underscore the need for increased investment, strengthening and scale-up of peer-mother adherence support services in PMTCT care across high HIV-burden countries in Africa.

## Supporting information

S1 Table The adjusted hazard ratios of ART attrition after enrolment in PMTCT care, by study arm and other baseline characteristics, among women studied. (DOCX)

## References

[1] World Health Organization. Programmatic update: Use of antiretroviral drugs for treating pregnant women and preventing HIV infection in infants, executive summary. In: Geneva: WHO; 2012: http://www.who.int/hiv/PMTCT_update.pdf. Accessed 4 October 2015.

[2] World Health Organization. Guideline on When to Start Antiretroviral Therapy and on Pre-Exposure Prophylaxis for HIV. In: Geneva: WHO; 2015: https://apps.who.int/iris/bitstream/handle/10665/186275/9789241509565_eng.pdf?sequence=1. Accessed 24 November 2019. 26598776

[3] Joint United Nations Programme on HIV/AIDS. Start Free, Stay Free, AIDS Free Final report on 2020 targets. In: Geneva: UNAIDS; 2021: https://www.unaids.org/sites/default/files/media_asset/start-free-stay-free-aids-free-2020-progress-report_en.pdf. Accessed 29 Oct 2021.

[4] Knettel BA, Cichowitz C, Ngocho JS, Knippler ET, Chumba LN, Mmbaga BT, et al. Retention in HIV Care During Pregnancy and the Postpartum Period in the Option B+ Era: Systematic Review and Meta-Analysis of Studies in Africa. J Acquir Immune Defic Syndr. 2018;77(5):427–438. doi: 10.1097/QAI.0000000000001616 29287029 PMC5844830

[5] Sibanda EL, Weller IV, Hakim JG, Cowan FM. The magnitude of loss to follow-up of HIV-exposed infants along the prevention of mother-to-child HIV transmission continuum of care: a systematic review and meta-analysis. AIDS. 2013;27(17):2787–2797. doi: 10.1097/QAD.0000000000000027 24056068 PMC3814628

[6] Coutsoudis A, Goga A, Desmond C, Barron P, Black V, Coovadia H. Is Option B+ the best choice? The Lancet. 2013;381(9863):269–271. 10.1016/S0140-6736(12)61807-8 23351797

[7] Ahmed S, Kim MH, Abrams EJ. Risks and benefits of lifelong antiretroviral treatment for pregnant and breastfeeding women: a review of the evidence for the Option B+ approach. Current opinion in HIV and AIDS. 2013;8(5):474–489. doi: 10.1097/COH.0b013e328363a8f2 23925003

[8] Van de Perre P, Tylleskar T, Delfraissy JF, Nagot N. How evidence based are public health policies for prevention of mother to child transmission of HIV? BMJ. 2013;346:f3763. doi: 10.1136/bmj.f3763 23788455

[9] Bailey H, Zash R, Rasi V, Thorne C. HIV treatment in pregnancy. The lancet HIV. 2018;5(8):e457–e467. doi: 10.1016/S2352-3018(18)30059-6 29958853

[10] Schmitz K, Basera TJ, Egbujie B, Mistri P, Naidoo N, Mapanga W, et al. Impact of lay health worker programmes on the health outcomes of mother-child pairs of HIV exposed children in Africa: A scoping review. PLoS One. 2019;14(1):e0211439. doi: 10.1371/journal.pone.0211439 30703152 PMC6355001

[11] Ambia J, Mandala J. A systematic review of interventions to improve prevention of mother-to-child HIV transmission service delivery and promote retention. Journal of the International AIDS Society. 2016;19(1):20309. doi: 10.7448/IAS.19.1.20309 27056361 PMC4824870

[12] Jopling R, Nyamayaro P, Andersen LS, Kagee A, Haberer JE, Abas MA. A Cascade of Interventions to Promote Adherence to Antiretroviral Therapy in African Countries. Current HIV/AIDS reports. 2020;17(5):529–546. doi: 10.1007/s11904-020-00511-4 32776179 PMC7497365

[13] Kisigo GA, Ngocho JS, Knettel BA, Oshosen M, Mmbaga BT, Watt MH. "At home, no one knows": A qualitative study of retention challenges among women living with HIV in Tanzania. PLoS One. 2020;15(8):e0238232. doi: 10.1371/journal.pone.0238232 32853233 PMC7451655

[14] Carbone NB, Njala J, Jackson DJ, Eliya MT, Chilangwa C, Tseka J, et al. "I would love if there was a young woman to encourage us, to ease our anxiety which we would have if we were alone": Adapting the Mothers2Mothers Mentor Mother Model for adolescent mothers living with HIV in Malawi. PLoS One. 2019;14(6):e0217693. doi: 10.1371/journal.pone.0217693 31173601 PMC6555548

[15] Ndaimani A, Chitsike I, Haruzivishe C, Stray-Pedersen B. An Exploration of Barriers and Enablers of Retention in a Program to Reduce Vertical Transmission of HIV at Health Centers in Zimbabwe. Int J Prev Med. 2019;10:74. doi: 10.4103/ijpvm.IJPVM_471_17 31198509 PMC6547947

[16] Gugsa S, Potter K, Tweya H, Phiri S, Sande O, Sikwese P, et al. Exploring factors associated with ART adherence and retention in care under Option B+ strategy in Malawi: A qualitative study. PLoS One. 2017;12(6):e0179838. doi: 10.1371/journal.pone.0179838 28636669 PMC5479573

[17] Klaus K, Baldwin J, Izurieta R, Naik E, Seme A, Corvin J, et al. Reducing PMTCT attrition: Perspectives of HIV+ women on the Prevention of Mother-To-Child HIV services in Addis Ababa, Ethiopia. Ethiop Med J. 2015;53(2):91–104. 26591297

[18] Wanga I, Helova A, Abuogi LL, Bukusi EA, Nalwa W, Akama E, et al. Acceptability of community-based mentor mothers to support HIV-positive pregnant women on antiretroviral treatment in western Kenya: a qualitative study. BMC Pregnancy Childbirth. 2019;19(1):288. doi: 10.1186/s12884-019-2419-z 31409297 PMC6693232

[19] Odiachi A, Al-Mujtaba M, Torbunde N, Erekaha S, Afe AJ, Adejuyigbe E, et al. Acceptability of mentor mother peer support for women living with HIV in North-Central Nigeria: a qualitative study. BMC Pregnancy Childbirth. 2021;21(1):545. doi: 10.1186/s12884-021-04002-1 34364384 PMC8349095

[20] Hamilton ARL, le Roux K, Young CW, Södergård B. Mentor Mothers Zithulele: exploring the role of a peer mentorship programme in rural PMTCT care in Zithulele, Eastern Cape, South Africa. Paediatr Int Child Health. 2020;40(1):58–64. doi: 10.1080/20469047.2018.1474697 30102134

[21] Vrazo AC, Firth J, Amzel A, Sedillo R, Ryan J, Phelps BR. Interventions to significantly improve service uptake and retention of HIV-positive pregnant women and HIV-exposed infants along the prevention of mother-to-child transmission continuum of care: systematic review. Trop Med Int Health. 2018;23(2):136–148. doi: 10.1111/tmi.13014 29164754

[22] Herce ME, Chagomerana MB, Zalla LC, Carbone NB, Chi BH, Eliya MT, et al. Community-facility linkage models and maternal and infant health outcomes in Malawi’s PMTCT/ART program: A cohort study. PLoS Med. 2021;18(9):e1003780. doi: 10.1371/journal.pmed.1003780 34534213 PMC8516224

[23] Phiri S, Tweya H, van Lettow M, Rosenberg NE, Trapence C, Kapito-Tembo A, et al. Impact of Facility- and Community-Based Peer Support Models on Maternal Uptake and Retention in Malawi’s Option B+ HIV Prevention of Mother-to-Child Transmission Program: A 3-Arm Cluster Randomized Controlled Trial (PURE Malawi). J Acquir Immune Defic Syndr. 2017;75 Suppl 2:S140–s148. 28498183 10.1097/QAI.0000000000001357

[24] Sam-Agudu NA, Cornelius LJ, Okundaye JN, Adeyemi OA, Isah HO, Wiwa OM, et al. The impact of mentor mother programs on PMTCT service uptake and retention-in-care at primary health care facilities in Nigeria: a prospective cohort study (MoMent Nigeria). J Acquir Immune Defic Syndr. 2014;67 Suppl 2:S132–138. doi: 10.1097/QAI.0000000000000331 25310119

[25] Tanzania National Bureau of Statistics, Zanzibar Office of Chief Government Statistician. 2012 Population and housing census, population distribution by administrative areas. In: Dar es Salaam: NBS; 2013: https://www.nbs.go.tz/nbs/takwimu/census2012/Census_General_Report.zip. Accessed 21 May 2021.

[26] Tanzania Commission for AIDS (TACAIDS), Zanzibar AIDS Commission (ZAC). Tanzania HIV Impact Survey (THIS) 2016–2017: Final Report. In: Dar es Salaam: TACAIDS; 2018: https://www.nbs.go.tz/nbs/takwimu/this2016-17/THIS_2016-2017_Final_Report.pdf. Accessed 20 October 2019.

[27] Routine health management information system data from the web-based Tanzania national district health information system (DHIS2). MOHCDGEC. https://dhis.moh.go.tz/dhis-web-commons/security/login.action. Accessed 10 November 2021.

[28] Smith Fawzi MC, Siril H, Liu Y, McAdam K, Ainebyona D, McAdam E, et al. Agents of change among people living with HIV and their social networks: stepped-wedge randomised controlled trial of the NAMWEZA intervention in Dar es Salaam, Tanzania. BMJ global health. 2019;4(3):e000946. doi: 10.1136/bmjgh-2018-000946 31179027 PMC6528754

[29] Cooperrider D, Whitney DD, Stavros J. The appreciative inquiry handbook: For leaders of change. Berrett-Koehler Publishers; 2008.

[30] Bandura A. Health promotion from the perspective of social cognitive theory. Psychol Health. 1998;13(4):623–649.

[31] Cleves M, Gould W, Gutierrez RG, Marchenko Y. An introduction to survival analysis using Stata, third edition. Stata press; 2010.

[32] Sam-Agudu NA, Ramadhani HO, Isah C, Anaba U, Erekaha S, Fan-Osuala C, et al. The Impact of Structured Mentor Mother Programs on 6-Month Postpartum Retention and Viral Suppression among HIV-Positive Women in Rural Nigeria: A Prospective Paired Cohort Study. J Acquir Immune Defic Syndr. 2017;75 Suppl 2:S173–s181. doi: 10.1097/QAI.0000000000001346 28498187

[33] Osborn L, Ronen K, Larsen AM, Richardson B, Khasimwa B, Chohan B, et al. Antenatal depressive symptoms in Kenyan women living with HIV: contributions of recent HIV diagnosis, stigma, and partner violence. AIDS Care. 2021:1–9. 10.1080/09540121.2021.1981216 PMC8758509 34579601

[34] Morojele R. Concerns about starting antiretroviral treatment among pregnant women in Lesotho. Global public health. 2021:1–14. doi: 10.1080/17441692.2021.1954225 34255609

[35] Lyatuu GW, Mwashemele SZ, Urrio R, Naburi H, Kashmir N, Machumi L, et al. Long-term virological outcomes in women who started option B+ care during pregnancy for prevention of mother-to-child transmission of HIV in Dar es Salaam, Tanzania: a cohort study. The lancet HIV. 2021;8(5):e256–e265. doi: 10.1016/S2352-3018(20)30308-8 33581776

[36] Dear N, Duff E, Esber A, Parikh A, Iroezindu M, Bahemana E, et al. Transient reductions in HIV clinic attendance and food security during the COVID-19 pandemic for people living with HIV in four African countries. Clin Infect Dis. 2021. 10.1093/cid/ciab379 PMC8135576 33906235

[37] World Health Organization. WHO Coronavirus (COVID-19) Dashboard. WHO. https://covid19.who.int/. Published 2021. Accessed 29 November, 2021.

