# Supplementary material for: Effect of peer-mother interactive programme on prevention of mother-to-child HIV transmission outcomes among pregnant women on anti-retroviral treatment in routine healthcare in Dar es Salaam, Tanzania
Source: PLOS Glob Public Health. 2022 Mar 9;2(3):e0000256. doi: 10.1371/journal.pgph.0000256 (PMC10021914; doi:10.1371/journal.pgph.0000256)
Supplement: S1 Table — (DOCX) [file pgph.0000256.s001.docx]

**S1 Table.** **The adjusted hazard ratios of ART attrition after enrolment in PMTCT care, by study arm and other baseline characteristics, among women studied**

| **Characteristic** | Multivariable, complete case^†^ | P value |
| --- | --- | --- |
| Peer-mother versus control arm | 0.85 (0.67, 1.08) | 0.18 |
| Maternal age, years |  | <0.0001 |
| <20 | 2.02 (1.50, 2.72) |  |
| 20-29 | 1.44 (1.24, 1.67) |  |
| 30-39 | 1 [referent] |  |
| 40+ | 0.93 (0.66, 1.32) |  |
| Gestational age, weeks |  | 0.0003 |
| <13 (first trimester) | 1 [referent] |  |
| 13-27 (second trimester) | 1.27 (1.04, 1.53) |  |
| ≥28 (third trimester) | 1.62 (1.28, 2.04) |  |
| Advanced vs early stage HIV* | 0.95 (0.77, 1.17) | 0.61 |
| ART status |  | <0.0001 |
| On ART before PMTCT care | 0.42 (0.35, 0.50) |  |
| Started ART at/after PMTCT care | 1 [referent] |  |
| Did not start ART | 12.89 (1.75, 95.13) |  |
| ART regimen backbone |  | 0.63 |
| NNRTI | 1 [referent] |  |
| PI | 0.93 (0.51, 1.67) |  |
| DTG | 0.84 (0.59, 1.20) |  |
| PMTCT volume, women/ year |  | 0.082 |
| 1-12 | 1 [referent] |  |
| 13-60 | 0.70 (0.50, 0.97) |  |
| 61-363 | 0.68 (0.46, 1.01) |  |
| ≥50% couple HIV testing coverage at ANC vs <50% | 0.78 (0.61, 0.99) | 0.042 |

^†^Cox proportional hazards regression with shared frailties adjusting for clustering in facilities and baseline imbalances after randomization (all variables on the table). Complete case N = 1,709 in peer-mother facilities and N = 1,172 in control facilities. Data in parentheses are 95% Confidence Intervals. *WHO stage III/ IV or CD4 count <200cells/µL

**Abbreviations:** PMTCT = prevention of mother-to-child transmission of HIV; ART = anti-retroviral treatment; NNRTI= non-nucleoside reverse transcriptase inhibitors; PI= protease inhibitors, DTG= dolutegravir. HIV = human immune deficiency virus; WHO = World Health Organization; CD = cluster of differentiation; ANC = antenatal care; vs = versus
